# Cell wall-deficient, L-form bacteria in the 21st century: a personal perspective

**DOI:** 10.1042/BST20160435

**Published:** 2017-04-13

**Authors:** Jeff Errington

**Affiliations:** Centre for Bacterial Cell Biology, Newcastle University, Newcastle-upon-Tyne NE1 7RU, U.K.

**Keywords:** bacteria, cell proliferation, cell wall, membranes

## Abstract

The peptidoglycan (PG) cell wall is a defining feature of the bacteria. It emerged very early in evolution and must have contributed significantly to the success of these organisms. The wall features prominently in our thinking about bacterial cell function, and its synthesis involves the action of several dozen proteins that are normally essential for viability. Surprisingly, it turns out to be relatively simple to generate bacterial genetic variants called L-forms that completely lack PG. They grow robustly provided that lack of the cell wall is compensated for by an osmoprotective growth medium. Although their existence has been noted and studied on and off for many decades, it is only recently that modern molecular and cellular methods have been applied to L-forms. We used *Bacillus subtilis* as an experimental model to understand the molecular basis for the L-form switch. Key findings included the discovery that L-forms use an unusual blebbing, or tubulation and scission mechanism to proliferate. This mechanism is completely independent of the normal FtsZ-based division machinery and seems to require only an increased rate of membrane synthesis, leading to an increased surface area-to-volume ratio. Antibiotics that block cell wall precursor synthesis, such as phosphomycin, efficiently induce the L-form switch without the need for genetic change. The same antibiotics turned out to induce a similar L-form switch in a wide range of bacteria, including *Escherichia coli*, in which we showed that proliferation was again FtsZ-independent. Aside from further basic science, future work on L-forms is likely to focus on their possible role in chronic or recurrent infections, their use as a model in studies of the origins of life, and possibly, biotechnological applications.

## The bacterial cell wall

The cell wall is an almost ubiquitous feature of the domain Bacteria. The major component of the cell wall is called peptidoglycan (PG), which comprises long glycan strands cross-linked by short peptide bridges [1]. The PG forms an elastic meshwork that covers the whole surface of the cell and which serves to protect the cell from damage, resist the outward turgor pressure due to the high osmolarity of the cytoplasm, and confer shape. The precursor molecule for wall synthesis, called lipid II, is made inside the cytosol. It is composed of a disaccharide of amino sugars, *N*-acetylglucosamine and *N*-acetylmuramic acid, carrying a short peptide side chain containing unusual d-amino acids. The precursor is linked to a special isoprenoid lipid carrier called bactoprenol to form lipid II. This is then flipped to the outer surface of the cytoplasmic membrane, where enzymes, called glycosyltransferases, polymerise the disaccharide moieties to form the glycan strands and transpeptidases, called penicillin-binding proteins, make bridges between adjacent strands [2]. A new family of putative glycosyltransferases, the RodA/FtsW (also called SEDS) proteins, was recently described [3,4] (see also [5]).

The shape of the cell is, to an extent, dictated by the shape of the PG layer. In many rod-shaped bacteria, such as *Bacillus subtilis* and *Escherichia coli*, an actin-like family of proteins, called MreBs, form polymers at the inner surface of the cytoplasmic membrane, where they are thought to organise the cell wall synthetic enzymes and provide spatial direction to cell wall synthesis, thereby governing cell shape [6]. Another important ‘cytoskeletal’ protein, FtsZ, of the tubulin superfamily plays a similar role in governing PG synthesis during cell division [7,8]. Some rod-shaped bacteria lack MreB proteins and use a different growth strategy in which PG synthesis and remodelling occur at the tip of the rod [9,10]. Coccoid bacteria have yet other strategies for controlling their shape and wall synthesis, but virtually all bacteria have PG as their main cell wall shape-determining component [11].

## Applying modern molecular cell biology methods to the L-form problem

In the mid-2000s, my laboratory became interested in an old problem relating to the apparent existence of bacterial variants that are capable of living in a cell wall-free state, called the L-form (or L-phase). There was an extensive literature on L-forms, going back to the 1930s [13,14], largely based on hospital case histories of patients with infections refractory to treatment with β-lactam antibiotics, or challenge studies in which animals were infected with walled or L-form cells, and progression of infection or clearance of the bacterial cells was followed. In the early days, there was confusion about whether L-forms should be distinguished from pleuropneumonia-like organisms (PPLOs). PPLOs are now called mycoplasmas, which are wall-deficient bacteria that have undergone millions of years of evolution to adapt to the wall-deficient state. L-forms, in contrast, are now usually assumed to be closely related to walled bacteria and often are able to switch back to the walled state.

L-forms are pleomorphic and osmotically sensitive because of their cell wall defect. However, they are also completely resistant to a range of antibiotics that work on cell walls, and there were sporadic reports of L-forms being involved in a wide range of often chronic or recurrent infections (reviewed in refs [15,16]). The literature on L-forms was quite extensive, but it peaked around the late 1970s, just before the advent of DNA sequencing and other methods that would have made molecular studies of the L-form state more tractable. In ca. 2004–2005, we decided to revisit the L-form problem using modern molecular cell biology and genomic methods in our favourite laboratory organism, *B. subtilis*.

*B. subtilis* had been reported to be able to enter the L-form state in earlier laboratory work [16], as well as in environmental studies of plant–microbe interactions [17]. Richard Daniel, then a postdoc in my laboratory, acquired an environmental L-form isolate of *B. subtilis* from a laboratory in Aberdeen (that of Eunice Allan; [18]) and began investigating its properties. Working with the strain was frustrating because it was tricky to grow (e.g. requiring osmotically supportive medium) but also because our attempts to introduce fluorescent (GFP) markers or other genetic changes that would help us to study its properties could not be achieved by our standard genetic manipulation methods. The classic laboratory strain of *B*. *subtilis* is attractive as a model because it is extremely amenable to genetic transformation, but other environmental isolates are often not so tractable. Nevertheless, imaging of the ‘naked’ L-forms revealed a startling degree of morphological complexity, including long strands of cytoplasm joining adjacent pleomorphic cells, so we were encouraged to continue with the project. A couple of years later, after my laboratory had moved from Oxford to Newcastle University, a finishing PhD student, Mark Leaver, wished to stay on for another year to carry out some high-risk, high-reward experiments and became interested in the L-form project. With Richard, he spent a few frustrating months trying to work out how to generate L-forms from *B. subtilis*. L-forms are very slow growing and are rapidly outgrown by walled cells. So, classical L-form protocols often rely on the presence of antibiotics such as penicillin to select for L-forms and prevent the growth of walled cells. However, it turned out that, at least for *B. subtilis*, the presence of penicillin actually blocks the initial generation of L-forms, for reasons that are only becoming clear now (Kawai et al., in preparation). Mark eventually succeeded in forcing *B. subtilis* to make the L-form switch [19]. He took advantage of a strain that Richard had made in which the genes for cell wall precursor formation could be turned on or off depending on the presence of an inducer, xylose (*P_xyl_-murE*). Repression of wall synthesis in this strain, by withholding xylose, forced *B. subtilis* into a wall-deficient state, provided that they also had an osmoprotectant (in this case sucrose) to prevent cell lysis. The key to the protocol was to select with penicillin later, after the cells had the chance to switch into the L-form state, following which they appeared to be able to grow indefinitely. We also played around with some genetic tricks, such as having a second copy of the xylose repressor gene in the cells, to prevent mutants capable of making cell wall in the absence of xylose from emerging and taking over the plates. Once this protocol had been developed, Mark found that he could select for L-form growth in any of our genetically manipulated strains [19].

It was clear from the frequency at which the L-forms emerged that at least one mutation (in addition to repression of *P_xyl_-murE*) was needed to enable the cells to grow. Attempts to map that mutation were frustrating because the L-forms were not easy to manipulate. However, we were fortunate that the timing of the project coincided with the emergence of whole genome sequencing. Newcastle had just set up a facility to do this and Jonathan Coxhead helped us to obtain the sequence of an L-form variant. After a considerable amount of bioinformatics, Mark was able to identify a single-point mutation that was present in the L-form compared with its parent strain. The mutation lay in a gene called *ispA*, encoding geranyl-geranyl pyrophosphate synthase, which is conserved from bacteria to man [20]. It is required for synthesis of polyprenoid lipids. The mutation was a single base substitution, but it altered a residue that had been shown in the rat enzyme to virtually eliminate function. Mark showed that the mutation was sufficient to enable L-form growth when cell wall synthesis was shut down, but it was several years before we worked out precisely what the mutation did. In fact, at the time, we did little to follow up on this result because it seemed obvious from metabolic maps that *ispA* should be required for making the carrier molecule, bactoprenol, on which PG precursors are assembled. Since we were blocking another (later) step in precursor synthesis, we assumed that the *ispA* mutation prevented accumulation of a toxic intermediate or compensated for some kind of metabolic imbalance that occurs when PG precursor synthesis is shut down. However, this turned out not to be the whole story (see below).

## Proliferation without a division machine

The L-forms had, as expected, the highly pleiomorphic shapes described in earlier literature and seen in our earlier experiments with environmental L-forms. They also had a huge range of sizes. Part of Mark's motivation for developing L-forms had been to ask a fundamental question about the function of the central player in bacterial cell division, FtsZ. FtsZ forms a ring-like structure at the site of impending cell division, where it also recruits various proteins required for cell wall synthesis [8]. We did not know whether the Z-ring worked directly to drive constriction of the cell membrane at the division site, or whether it simply recruited division proteins, including cell wall synthases, which contributed the constrictive force. We anticipated being able to answer this question in L-forms because of their lack of cell wall function. With Mark's protocol, we could make L-forms from any of our genetically manipulated strains, so one of the first things that Mark did was to make L-forms from a strain bearing an FtsZ–GFP fusion, so that we could look at the Z-rings. It turned out to be quite difficult to visualise the GFP fusion because of the heterogeneous size and shape of the L-forms, their fragility, and our inability to immobilise them, so that they would stay in a focal plane. Nevertheless, the experiments suggested that L-forms rarely assemble the regular ring-like FtsZ structures of walled cells. We then started to wonder whether the L-forms used FtsZ at all to divide, so Mark built an L-form strain in which we could shut down expression of the *ftsZ* gene. In walled calls, this brings about a lethal cell division defect. The cells elongate without dividing, then become unstable and lyse. Remarkably, it seemed that repression of *ftsZ* expression made no difference in the viability or growth rate of our L-forms! One of the reviewers of our first L-form paper thought that this was such an important result that we needed to demonstrate that we could delete the *ftsZ* gene to prove that it was non-essential. This was technically challenging because of the essential nature of *ftsZ* in walled cells, but Mark was eventually able to build an L-form strain with a complete deletion of *ftsZ* (and the adjacent *ftsA* division gene for good measure), conclusively showing that the L-forms did not require the normal division machine [19]. We were astonished by this result because FtsZ was widely conserved across the bacterial domain, and essential for viability virtually everywhere it had been tested (*Streptomyces* being one notable exception; [21]). This was our first hint that the study of L-forms might turn out to be much more interesting and important than we had anticipated.

## An unexpected bizarre mode of proliferation

**Figure 1.**
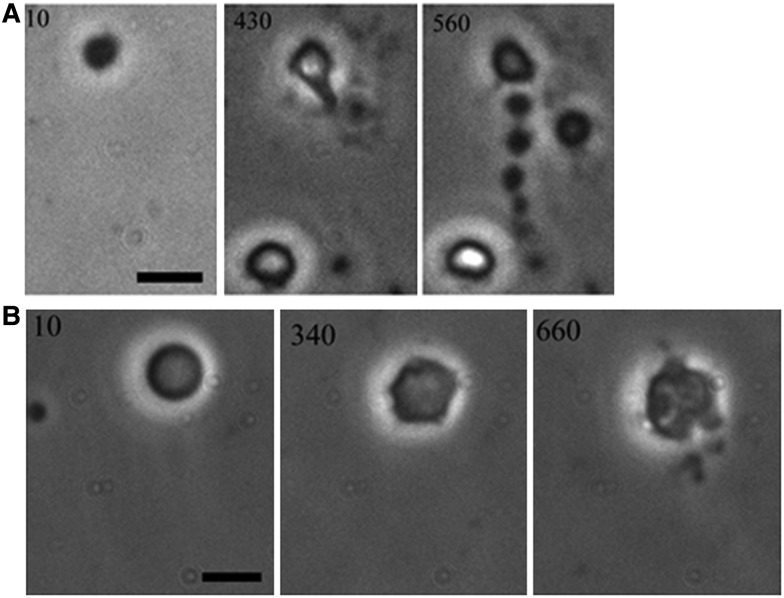
Examples of proliferative events in L-forms of *B.* subtilis, as viewed by phase contrast microscopy. Numbers refer to time (min) of observation (from ref. [19]). (**A**) An event we called extrusion–resolution. A spherical L-form increases in size, then a tubular protrusion emerges which breaks down into a chain of connected progeny cells. (**B**) A larger L-form again starts as a sphere, then undergoes pulsating changes in shape before multiple small progeny cells erupt from at least three different places on the cell surface.

The *ftsZ* result raised an important question about the nature of L-form proliferation. Long-term time lapse imaging of the L-forms turned out to be difficult for various technical reasons. Nevertheless, one Saturday morning I got an excited email from Mark. He told me that he had got the time lapse imaging to work and had seen proliferative events, but that they did not fit with any of our models. I excitedly waited for the movies to download and was amazed at what I saw. In fact, I went running around the house trying to find someone else to show — sadly, my daughter (then aged about 17) was not as excited as I was!. The most prominent event captured in the movie (Figure 1A) was a cell, more or less round, which, over several hours, grew in size before elaborating a protrusion, which grew into a long tube that then resolved into a chain of what appeared to be progeny cells, which appeared to remain connected by tiny tubular connections. Another larger L-form showed a somewhat different behaviour (Figure 1B). To begin with, it had a more or less spherical shape but then, again over a period of hours, surface features, bulges, and dimples appeared at multiple places on the surface. This was followed by the eruption of multiple progeny across at least three different sites on the cell. Thus, L-forms clearly did not follow the binary fission process that typifies almost all cells that have been described. The startling new findings on loss of requirement for FtsZ and the bizarre mode of proliferation, which we termed ‘extrusion resolution’, were published in a full article in *Nature*, which coincided with the celebration of Darwin 200 [19]. I later mused that Darwin would have been interested in the identification of a possible early intermediate step in the evolution of life.

## Nothing is new under the sun

A little while after the paper was published I received a typewritten letter from Gertrud and Otto Kandler. The letter was generally complimentary but it contained a wry sentence. ‘We are delighted to see that the L-forms still replicate by the mechanism described in our paper of 1954’. We rushed off to find a copy of the paper [22] and found that it did indeed contain wonderful phase contrast images similar to the ones we had just published. The paper had been written in German, did not contain the key word ‘L-forms’, and had only been cited a handful of times, finally in the 1970s, so we did not feel too bad about having missed it. We published a corrigendum in *Nature* pointing to the earlier paper. Of course, in 1954, nothing was known about the FtsZ ring machinery, or even about the structure of the cell wall, and the nature or origins of the organisms described by the Kandlers were not well understood. Nevertheless, the story makes clear that it is very difficult to be completely original in science!

## Reversible L-forms

Patri Domínguez-Cuevas and Romain Mercier joined the laboratory, respectively, just before and just after Mark Leaver moved on, and they took up the mantle of the L-form work. I also received an ERC Advanced Grant to study the problem further. Patri made new L-form strains and discovered one that switched efficiently back and forth between the L-form and walled state [23]. By sequencing this organism, we discovered that the emergence of L-forms from walled cells requires, at least under some conditions, e.g. penicillin selection, mutations that facilitate escape of the membrane-bounded L-form from the cylindrical cell wall. One regulatory mutation probably works by counteracting penicillin-triggered defence mechanisms that prevent or limit the degradation of the wall, and which would lead, under non-osmotically protective conditions, to cell lysis. The second mutation affected a cell division gene *sepF*, mutation of which leads to malformed division septa [24] that can apparently fracture, leading to L-form escape [23].

## Membrane fluidity and its importance in L-form progeny scission

**Figure 2.**
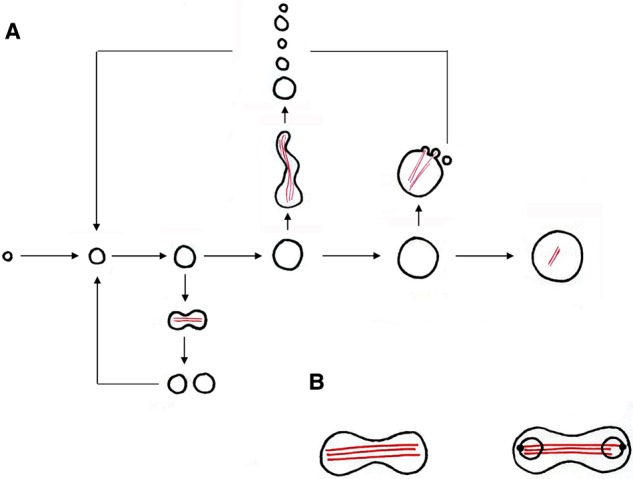
Schematic drawing (by Mark Leaver) of our early ideas on possible mechanisms for L-form proliferation. (**A**) The central path illustrates that L-forms have a wide range of sizes. After a period of growth, proliferation can take any one of many forms, from essentially binary fission to the proliferative events exemplified in Figure 1. Red lines illustrate hypothetical cytoskeletal filaments that could be involved in driving shape changes leading to proliferation. (**B**) Model for proliferation based on the idea that active segregation of chromosomes (illustrated again by putative cytoskeletal or motive filaments) could drive shape changes leading to proliferation.

In these early days, we were convinced that the amazing extrusion resolution process must be driven by proteins. Figure 2 shows an early model in which we assumed that cytoskeletal fibrils (red lines), potentially of MreB, would drive the shape changes responsible for division. In Figure 2B, we imagined alternative models in which mechanisms responsible for segregation of chromosomes (which still remain unclear) might drive formation of the membrane protrusions. This class of model had the advantage that it would ensure that L-form progeny efficiently acquire the genetic information needed to propagate. Cytoskeletal proteins, such as *mreB*, seemed good candidates for proteins capable of driving the protrusions. However, extensive candidate gene knockout studies failed to identify factors needed for L-form growth [25]. These included making L-forms from a triple knockout of all three *mreB* paralogues of *B. subtilis* [26], as well as genes such as *divIVA*, encoding a versatile protein required in different ways for polar morphogenesis in a range of Gram-positive bacteria [27], or chromosome segregation genes (in case segregation drove blebbing or tubulation). However, availability of a mutant that could readily switch between states enabled us carry out an unbiased genetic screen [25]. Patri and Romain eventually found a mutant that could grow normally in the walled state but was completely unable to grow as an L-form. Genome sequencing of the mutant revealed that it had a point mutation probably inactivating a gene called *bkd* that was required for branched-chain fatty acid synthesis. Romain showed that the mutation probably worked directly on enzyme activity because he could restore growth to the mutant by providing the branched-chain fatty acid precursors that would be lacking in the mutant. This provided the first hint that properties of the cytoplasmic membrane might be important for L-form growth. Romain showed that the primary effect of the mutation was to reduce anteiso-branched-chain fatty acids, which increase membrane fluidity relative to the closely related iso-forms. Morphological characterisation of the mutant showed that the L-forms could grow for a significant period of time, and undergo changes in shape, but they did not resolve into separate progeny. We concluded that the mutant was affected in the final step of division, which we called scission, and that a relatively high level of membrane fluidity was required for this process [25].

## A simple biophysical mechanism for L-form proliferation

Romain Mercier teamed up with another geneticist, Dr Yoshi Kawai, to carry out an extensive genetic analysis of L-form growth [28]. All of the experiments we had done so far were based on repression of cell wall precursor synthesis (Richard Daniel's *P_xyl_-murE* construction). They wished to find out whether there were other ways to trigger L-form growth. They therefore took a strain with an *ispA* mutation and developed a way to screen for L-form colonies in this background. They also wished to find mutations that did not simply abolish wall precursor synthesis (like the *P_xyl_-murE* construct), so they looked for L-forms that could resume growth as walled cells. Among the mutations they found, one stood out — a mutation just upstream of the *accDA* operon, which encodes the catalytic subunit of acetyl-CoA-carboxylate synthase. This enzyme catalyses the key committing step in fatty acid synthesis. Yoshi showed that the mutation worked by increasing synthesis of the proteins and that it could be mimicked by a construction that enabled overproduction of the proteins via a second copy of *accDA* controlled by *P_xyl_*, elsewhere in the chromosome. Induction of *P_xyl_-accDA* in the presence of an *ispA* mutation induced L-form growth just as efficiently as repression of *P_xyl_-murE*. From previous work on fatty acid synthesis in *B. subtilis* [29], it was anticipated that increased acetyl-CoA-carboxylase synthase would increase the accumulation of malonyl-CoA, which would in turn induce the expression of various genes required for fatty acid synthesis, leading to increased membrane lipid accumulation. Furthermore, repression of PG precursor synthesis (as in our original L-form experiments with *P_xyl_-murE*) also led indirectly to increased membrane synthesis, by a mechanism that is still unclear, as did various other mutations that emerged from our genetic screens. These results led us, originally with a certain degree of scepticism, to the notion that L-form growth might simply be promoted by excess membrane synthesis [28].

The main comfort for us was that there turned out be both theoretical and practical support for the model [30]. Thus, Peter Walde's laboratory, in particular, had described experiments in which a lipid vesicle was induced to undergo L-form-like replication simply by ‘feeding’ it with fatty acids. By intercalating into the surface of the vesicle, the fatty acids increase its surface area to volume ratio, which is sufficient to generate ‘baby’ vesicles [31]. An exciting outcome of these experiments was that they showed that L-forms might provide an interesting model for the replication of primitive cells, way back before the evolutionary emergence of the cell wall [11,32].

## The L-form as an experimental tool for studying cell wall synthesis and cell division

L-forms are useful as a model for the origins of life, but they also provide powerful experimental systems for studying certain functions that are normally essential but become non-essential in the L-form state, such as the cell wall synthetic system itself and the FtsZ-based division machinery. We took advantage of this to study a long-standing question about whether cellular form requires a pre-existing template. This had long been debated [33], and it was a feature of certain models for cell wall synthesis, such as the 3-for-1 model of Holtje [34]. In the latter model, an existing glycan strand in the wall is hydrolysed and replaced with a ‘triple pack’ of new strands. Yoshi Kawai tested the formal requirement for a cell wall template by making L-forms in which PG precursor synthesis was prevented, by deletion of the essential *murC* gene, and then, after a period of growth in the absence of PG, transformed the cells with a plasmid carrying the *murC* gene, which resulted in restoration of normal growth and rod-shaped form. Thus, we were able to conclusively reject models for cell morphogenesis that require a pre-existing template [35].

## Generalisation of L-form principles to other bacterial groups

By about 2013, we were happy that we had achieved a relatively good understanding of the general principles underlying the walled to L-form transition and L-form growth in *B. subtilis*. However, it seemed important to explore whether these principles could be extended to other groups of bacteria. We could easily test whether inhibition of cell wall precursor synthesis might elicit the L-form switch by taking advantage of antibiotics, such as phosphomycin and d-cycloserine, that inhibit enzymes in the precursor pathway. Romain Mercier took a range of organisms of different classes and tested whether the inhibitor, in the presence of osmoprotectant, could generate L-forms. He showed that *Corynebacterium glutamicum*, a high G + C Gram-positive actinobacterium, switched beautifully into an L-form that grew well in liquid culture medium, much like *B. subtilis* L-forms. Even *E. coli*, a Gram-negative bacterium, separated by perhaps 1 billion years of evolution from *B. subtilis*, could grow in an L-form state, albeit requiring a semi-solid matrix or agar plate surface for efficient growth [36]. Earlier work on *E. coli* had suggested that under at least some conditions, L-form-like cells required a residual level of PG synthesis [37], suggesting that they might differ, perhaps quite fundamentally, from the *B. subtilis* L-forms. However, on the basis of our *B. subtilis* results, we argued that a useful operational definition of the L-form state was ability to grow in the absence of the division machine, and Romain was able to take advantage of the powerful *E. coli* genetics to generate null mutations in various genes, including those for cell wall precursor synthesis and two different essential cell division genes, *ftsZ* and *ftsK* [36].

There have been a plethora of different kinds of conditions used to generate cells called L-forms and it may well be that, in different organisms, the extent to which they can survive and thrive with reduced levels of cell wall synthesis may vary. We therefore suggest that ability to proliferate in the absence of the normally essential FtsZ-based division machine is a useful operational definition for the L-form. It is clear that, at least for Gram-positive *B. subtilis* and Gram-negative *E. coli*, these organisms are intrinsically able to switch readily to a mode of proliferation that is independent of the normally complex and essential FtsZ-based machine.

Interestingly, one of the organisms that Romain examined, *Caulobacter crescentus*, an α-proteobacterium, resisted his attempts to force growth in the L-form state. We speculate that this may be due to the intricate dependence of the cell cycle of this organism on polar morphogenesis [38], which is presumably impacted badly by cell wall inhibition.

## Solving the *ispA* conundrum

In the course of carrying out a detailed genetic dissection of the *B. subtilis* L-form transition, Yoshi and Romain also looked for mutations different from *ispA* that could support L-form growth when PG precursor synthesis was blocked (e.g. by repression of the *P_xyl_-murE* construct). Most primary mutations of this class, which we termed class II, lay in or near the *ispA* gene. To avoid this, we introduced a second copy of the *ispA* gene, so that two mutational hits would be required to eliminate IspA function. The new mutations, which generally gave rise to weaker growth than *ispA*, mapped to a variety of different genes. However, many of them lay in genes involved in the respiratory chain and oxidative phosphorylation. Others would induce oxidative stress responsive genes, while a third group would affect glycolysis. These findings led us to propose that the mutations work by reducing oxidative damage [39]. In support of this idea, Yoshi showed that inhibition of cell wall synthesis resulted in up-regulation of oxidative stress responsive genes, and that this stress was reduced by the class II mutations, including *ispA*. We then realised that rather than blocking bactoprenol synthesis (see above), which would in any case be lethal in non-L-form cells, the *ispA* mutation might also reduce or block the synthesis of menaquinone, another isoprenoid lipid, and a component of the respiratory chain. In further support of a model in which oxidative stress is experienced by wall-deficient *B. subtilis*, Yoshi showed that growth in the L-form state could be stimulated without an *ispA* mutation by use of anaerobic conditions or by the presence of exogenous scavengers of reactive oxygen species (ROS) [39]. Similar results were obtained for *E. coli*, suggesting that the oxidative stress effect is broadly conserved. We currently favour a model in which a block in cell wall synthesis, and thus utilisation of sugar-phosphate intermediates, leads to increased flux through the TCA cycle. ROS are then generated as a by-product of the metabolism of molecular oxygen by the electron transport chain. We are currently investigating how the design of metabolism and intricate connections between PG precursor synthesis, fatty acid synthesis, and other outputs of glycolysis combine to generate the above effects.

## Future challenges

The L-form project has ramified over the last 10 years to generate at least four different areas of interest. First, we still do not fully understand the basic biology of the L-form state. It is not completely clear how blocking cell wall precursor synthesis, stripping the cell wall (without blocking synthesis), or overproducing membrane, all elicit oxidative stress. The fact that this occurs in both Gram positives and negatives suggests that there are common principles in the design of metabolism that have been conserved over immense evolutionary time. The generation of oxidative stress by various antibiotics has been a controversial topic over the last few years [40] and further studies of L-forms may contribute significantly to the understanding of this complex area.

A second major topic of interest lies in the use of L-forms to inform about possible mechanisms for early steps in the evolution of cellular life [11,32]. The bacterial cell wall appears to be very ancient, possibly dating back to the earliest bacterial cells. Indeed, it is plausible that invention of the cell wall was a key step in enabling the bacterial radiation, providing the ability to withstand adverse changes in osmolarity, to achieve a defined shape and an efficient, tightly regulated division process [11]. Comparative studies of L-form proliferation and the replication of simple lipid vesicles are likely to be an interesting and informative area, identifying also the minimal requirements for proliferation.

Minimal cells are also of interest in biotechnology, in principle, providing a way to reduce the metabolic energy that could otherwise be directed towards biosynthesis of commercial products. Elimination of the wall could also provide a way to remove a potential barrier to the secretion of proteins and avoid synthesis of wall fragments that have potentially toxic immunostimulatory effects.

Finally, many key questions still remain in relation to the possible role of L-forms in all kinds of infectious diseases [14,15,41]. Now that we have a much better understanding of the molecular and physiological changes that accompany and promote L-form growth, we are in a strong position to revisit questions about L-forms in disease. We are presently engaged in various collaborations aimed at identifying L-forms or L-form-like cells in various disease states. Ongoing work appears very promising, but the ‘killer’ experiments that will finally generate the data to convince sceptical infectious disease clinicians remain tantalisingly out of reach. Please watch this space!

## Abbreviations

PG: peptidoglycan
PPLOs: pleuropneumonia-like organisms
ROS: reactive oxygen species.
